# Salvage Liver Transplantation Leads to Poorer Outcome in Hepatocellular Carcinoma Compared with Primary Liver Transplantation

**DOI:** 10.1038/srep44652

**Published:** 2017-03-15

**Authors:** Yuhua Shan, Lifeng Huang, Qiang Xia

**Affiliations:** 1 Department of Liver Surgery, Ren Ji Hospital, School of Medicine, Shanghai Jiao Tong University, Shanghai, China.

## Abstract

Hepatocellular carcinoma is the most common liver malignancy. Salvage liver transplantation (SLT) is viewed as a feasible cure for recurrence of HCC after resectomy, but the effect is under dispute. A retrospective study examined data at Renji Hospital for 239 transplants from January 2006 to December 2015, including 211 who received primary liver transplantation (PLT) and 28 who underwent SLT. A multivariable cox regression model was employed to pick out relative factors to overall survival (OS) and recurrence free survival (RFS). Propensity score matching (PSM) was used to balance the bias. Both OS and RFS were worse in SLT group than in PLT group, especially for those patients within Milan criteria. Our study demonstrates that SLT bears higher risk of recurrence and death than PLT, indicating that SLT should be given a more careful thought at performance.

Hepatocellular carcinoma (HCC) is the most common malignant tumor in liver known for its insidious onset and poor prognosis. Hepatic resection has remained the first-line choice to treat this tumor. Though, with the aid of other palliative and expectant treatments, the 5-year OS has reached 30–50%, the 5-year recurrent rate is still up to 70–85%^1^. In carefully selected patients, radical hepatectomy can be performed to cure the malignancy, with 5-year OS over 60%, and RFS averagely 40% or better^2^. Unfortunately, resection is only fit for a very small range of HCC. Some patients have beyond the criteria ideal for radical resectomy, with spread of tumor or invasion of major vessels (the portal vein, inferior vena cava (IVC) or the three main hepatic veins). Others suffering severe underlying cirrhosis haven’t enough residual liver to provide adequate functional reserve (<25% of a normal liver or <40% of a cirrhotic liver). Liver transplantation is therefore viewed as an important curative alternative for a relatively wider range of HCC patients. In order to achieve better survival, LT was at first considered to cover only those early-stage HCC patients with severe cirrhosis, and thus 5-year survival was encouragingly over 70%^3^. But in recent years, the indication was extended to those recipients over Milan criteria. In fact, there are no worldwide recognized selection criteria for LT. Due to the invasive and metastatic nature of HCC, recurrence is still the main problem after liver resection^4^. Patients with intrahepatic recurrence would be given an accurate assessment, and considered for salvage liver transplantation (SLT)^5^. The indications and selection criteria for SLT are still controversial. When compared with primary liver transplantation (PLT), different study came to contradictory conclusions. A bundle of researches reported that SLT has equal outcome to PLT^5^,^6^,^7^. Conversely, the outcome of SLT is suspected to be deteriorated by the greater invasive potential of tumors represented by shorter relapse-free survival (RFS) and more surgery times^8^,^9^. With regard to the shortage of organ donor, it must be a priority to validate and precise the indication of SLT so as to make more reasonable and appropriate distribution of the precious organ resource. The objective of this study is to evaluate the overall survival (OS) and RFS between patients who underwent PLT and SLT at our single center.

## Material and Method

### Patient Inclusion Criteria

**Figure 1:**
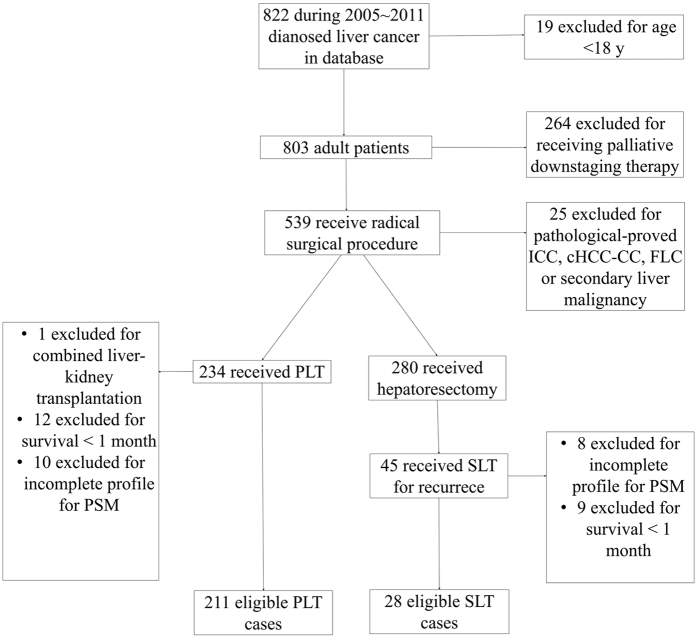
Patient flow diagram.

Approval was received from the Ethical committee of Renji Hospital Shanghai Jiaotong University School of Medicine, and each patient involved signed informed consent for reference to his clinical files. All methods employed in this study were performed in accordance with the relevant guidelines and regulations. Retrospectively, we reviewed the clinicopathological data on patients who underwent LT for HCC between 2005 and 2011 at the Renji Hospital Shanghai, China. SLT here is defined as LT performed in recurrent HCC patients who received previous radical resection. Those excluded according to flowchart in Fig. 1 were: patients without histological confirmation of HCC in the resected livers; patients with concurrent other malignancies, or with pathologically-confirmed other tumor types in removed samples, including interhepatric cholangiocellular carcinoma (ICC), combined hepatocellular carcinoma (cHCC-CC), Fibrolamellar hepatocellular carcinoma (FLC) and secondary metastatic tumor; patients received palliative downstaging therapy (resectomy, hepatic arterial chemoembolization, radiofrequency ablation etc.); patients died within 1 month after surgery; pediatric patients (<18 years); patients who received liver transplantation to treat liver function deterioration after resections. Among the 239 patients finally included in this retrospective study, 211 received PLT and 28 were recurrent cases who received radical hepatectomy before and then treated by LT protocol.

### Perioperative Assessment

Parameters compared between PLT and SLT are: age, sex, donor type, serum α fetal protein (AFP) level, Carbohydrate Antigen 19–9 (CA19–9) level, positive rate of hepatitis B surface antigen, hepatitis B (HBV) DNA and hepatitis C virus (HCV) RNA, CHILD-PUGH Score, tumor feature information such as tumor DIAMETE, number and metastasis. In this study, CHILD-PUGH Score was recognized as an index reflecting liver functional reserve.

### Surgical Procedure and Postoperative Management

In all the surgeries, 82.8% of the donors were deceased donors and 17.2% were living donors. Only prior hepatectomies with R0 margin status were considered radical and then, recruited in this study. All the transplantations were performed following standard techniques by experienced specialists in the Department of Liver Surgery, Ren Ji Hospital, Shanghai, China. Classic orthotopic LT was the only surgery type in the cadaveric donor group. All LDLT cases were operated on using right liver grafts without the middle hepatic vein. Biliary tract anastomosis was performed in a duct-to-duct form. The largest diameter of tumor as well as the number of tumor will be verified on the sample. Then, with the help of pretransplantation imaging information, all cases were classified as either within Milan criteria or beyond it. After LT, the primary immunosuppressive therapy involved tacrolimus (FK506) or cyclosporine (CsA) combined with methylprednisolone and mycophenolate mofetil (MMF). Steroids were withdrawn in 3 months. 41.8% of the patients received Rapamycin immunosuppression in later treatment. Cases with HBcAb and/or HBeAb positive, which indicating a potential or history HBV infection, all received prophylactic antivirus therapy. For those who still had HBVsAg and/or HBV DNA positive before transplantation, intravenous anti-HBV globulin was added after surgery.

### Outcome Definition

RFS was defined as the interval between surgery and recurrence. If recurrence didn’t happen, patients were censored at death, retransplantation or the date of the latest follow-up. The OS was calculated from the date of operation to the date of death or retransplantation. Patients who were alive at latest follow-up were treated as censored. The definition of recurrence includes imaging evidence of both recurrent tumor mass in liver or elsewhere, ascites or pleural effusion proved malignant by biopsy and elevating AFP alone without tumor finding.

### Statistical Analysis

Mean ± SD or median (interquartile range [IQR]) was used for description of continuous variables and No. (%) was for categorical factors. Difference between groups was assessed by Student t test for parametric variables and Mann-Whitney U test for nonparametric ones. A χ^2^ test was used to generate the univariate models describing the association of variables with OS or RFS. Variables with p values < 0.2 by univariate analysis were chosen for multivariate analysis by the use of a cox regression model. To reduce potential bias in this retrospective study, propensity-score based matching analysis (PSM) was employed, which included all possible variables. We performed caliper matching within a range of 0.2 multiplied by the standard deviations on the PS logit^10^. A Kaplan-Meier plot was constructed and log-rank test was adopted to compare the survival. A P value of < 0.05 was considered to be statistically significant. All analyses were performed with the aid of SPSS version 18.0.

## Results

Overall and propensity-score-matched Patients’ characteristics were listed in detail in Table 1. Of the total 239 patients, 211 were de novo HCC cases and 28 were recurrent cases. There was imbalance in some variants between the two LT groups (Table 1). The median pre-transplantation CA19-9 level was 36.7 U/mL (range, 18.60~56.90 U/mL) in PLT group and 20.4 U/mL (range, 8.50~37.15 U/mL) in SLT group respectively (p < 0.001). 92.9% of the entire patients had HBsAg positive, 56.9% still had HBV DNA proliferation active at the time of LT. The DNA positive rate was 63.03% in PLT group significantly higher than 32.14% in SLT group (p = 0.001). PLT group also had a worse liver functional reserve before LT (Child-Pugh A 36.02% compared with 60.71%, p = 0.026). No other characteristics showed significant differences between groups.

PSM of all possible variants generated 23 matched pairs of PLT and SLT. No significant differences were found between matched cases (Table 1).

Factors recruited in multivariate model are listed in Tables 2 and 3. According to cox regression model, factors associated with RFS and OS include serum AFP level, tumor mass and Milan stage (Tables 2 and 3). Rapamycin didn’t manifest significant effect on OS or RFS.

**Figure 2:**
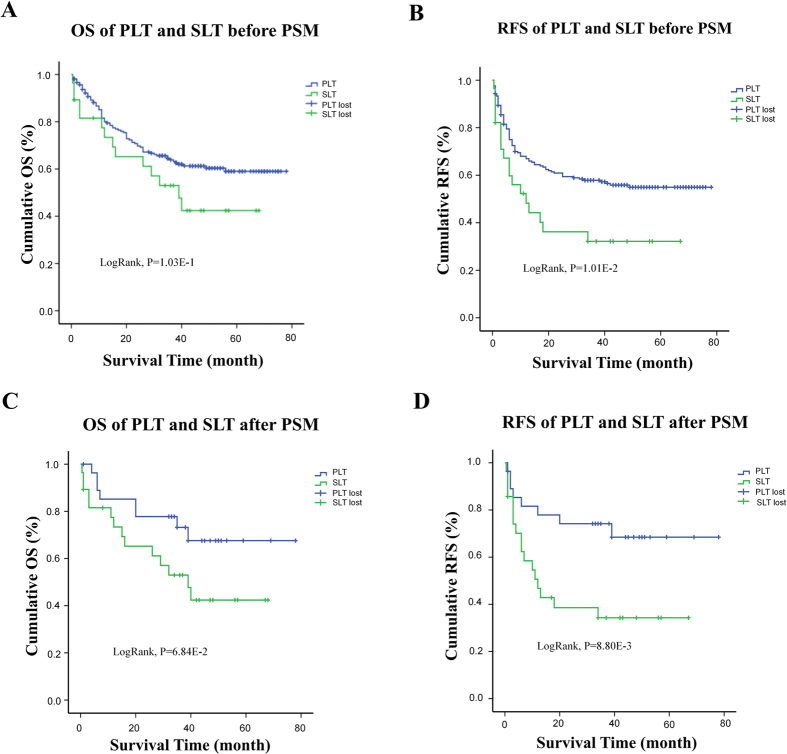
OS and RFS comparison between PLT and SLT group both before and after PSM. (**A**) and (**B**) OS and RFS both have an inferior prone in SLT group than in PLT group, with significance in RFS (LogRank p < 0.05) but not in OS (LogRank p > 0.05). (**C**) and (**D**) After PSM, the RFS seem significantly worse in SLT group. In a 1:1 match of 23 pairs, the 1 yr, 3 yr and 5 yr OS rate was 77.89%, 74.18% and 68.47% respectively in PLT group, significantly better than 62.34%, 34.29% and 34.29% in SLT group (LogRank P < 0.05). The OS phenomenon was in the same trend with an almost significance, in a 23 to 23 PSM, with 86.96%, 52.17% and 17.39% respectively in P group and 56.52%, 30.43% and 4.35% in SLT group (LogRank P > 0.05).

The entire median follow-up was 35 months (range, 11~52 months). The entire median follow-up was 35 months (range, 11~52 months). The entire median OS was 35 months, with the 1 yr, 3 yr and 5 yr OS respectively 80.00%, 63.86% and 59.02% in PLT group while 65.22%, 52.99% and 42.39% in SLT group. The entire median RFS was 32 months. The 1 yr, 3 yr and 5 yr RFS were 66.96%, 57.86% and 54.95% significantly better in PLT group than 48.25%, 32.17% and 32.17% in SLT group (Table 4; Fig. 2B). No significance was found between two groups in OS (P > 0.05, Table 4), but a trend of poorer outcome can be seen from Kaplan-Meier survival curves (Fig. 2A). On multivariate analysis, SLT is significantly associated with poorer RFS (HR 1.98, 95% CI 1.05–3.72, p = 0.035) (Table 4). Its unfavorable effect on OS is also significant (HR 2.17, 95% CI 1.18–4.01, p = 0.013) (Table 4).

After adjusted by PSM, the median follow up was 36 months (range, 12.0~52.75 months). The 1 yr, 3 yr and 5 yr RFS rate were 77.89%, 74.18% and 68.47% respectively in PLT group, significantly better than 62.34%, 34.29% and 34.29% in SLT group (P < 0.05). The OS was in the same trend, with 1 yr, 3 yr and 5 yr ratio of 85.19%, 73.20%, 67.58% respectively in PLT group and 73.38%, 52.99% and 42.39% in SLT group with an almost significance (Fig. 2C and D). When compared through multivariate Cox regression, SLT turned out to be a significantly unfavorable factor for RFS (OR = 3.53, 95% CI = 1.02–12.20, p = 0.037) and OS (OR = 2.84, 95% CI = 1.06–7.67, p = 0.038) (Table 4).

**Figure 3:**
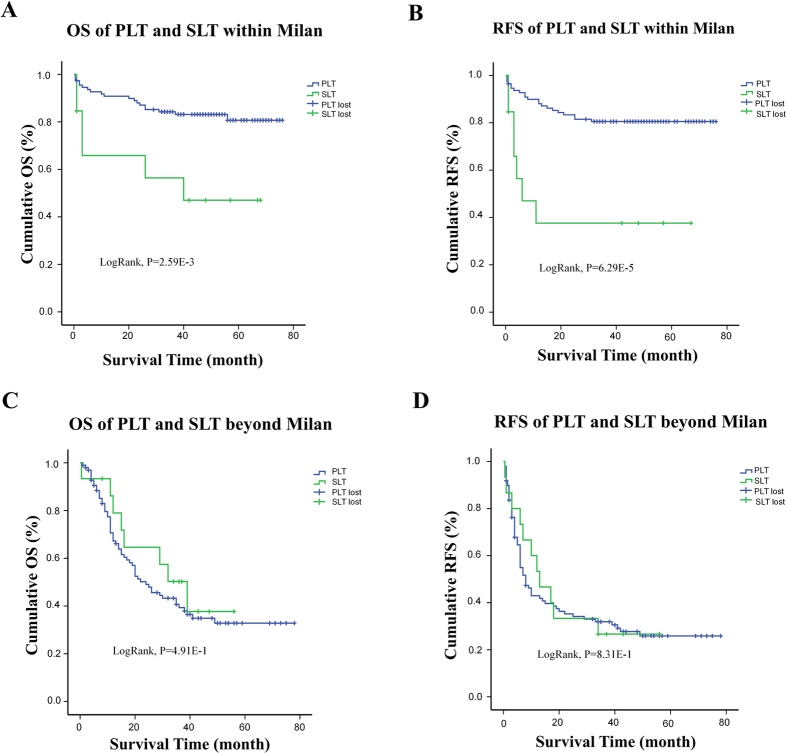
OS and RFS comparison between PLT and SLT group after split by Milan criteria. (**A**) and (**B**) For those within Milan stage, OS and RFS both have a inferior significance in SLT group than in PLT group (LogRank p < 0.05) (**C**) and (**D**) For those beyond Milan criteria, both OS and RFS had no significant differences between two groups.

**Figure 4:**
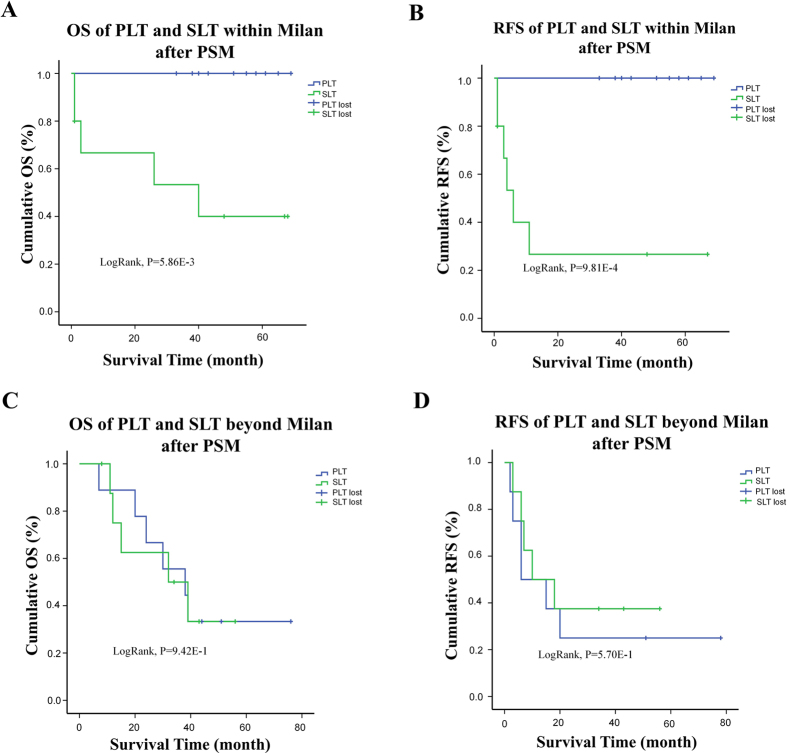
OS and RFS comparison between PLT and SLT group after split by Milan criteria with the use of PSM. (**A**) and (**B**) The significance of deteriorated OS and RFS in SLT group remained after PSM in within Milan group(LogRank p < 0.05) (**C**) and (**D**) After PSM, the beyond-Milan group still had no differences between SLT and PLT.

If we set apart those within Milan criteria (n = 126) and those who exceed the criteria (n = 113), we will find interestingly that SLT is a significant discordant factor on both OS and RFS in patients within Milan criteria (Fig. 3A and B). When matched by propensity scores of all the possible factors as mentioned before, gap between SLT and PLT become even deeper. After PSM, the 10 matched PLT patients had a median follow up time of 53 months compared to 14.5 months of their SLT counterpart, and all remain alive without recurrence at the end of follow up. SLT patients had a recurrence rate at 30% with median RFS at 3.5 months (Fig. 4A and B). For those beyond Milan criteria, no significant difference was told between two groups. 50% of the cases obtained recurrent tumor at around 9 months after transplantation, 50%of them died before the 26^th^ month after surgery (Figs 3C,D and 4C,D).

## Discussion

Though, The 5-year overall survival of HCC has reached 75% after liver transplantation^11^, against the background of organ shortages, improvements in the prognostic tools for predicting outcomes after LT for HCC are necessary. In our study, we found that both OS and RFS were significantly worse in SLT groups than in PLT group, when compared under a PSM model. This result is contrast to most of the conclusion in other researches. Such divergence may credit to a variety of factors.

Generally, different method in measurements and statistical analysis can come to different conclusion. Over the past decades, restrictive Milan criteria has been adopted to select HCC patients for LT. LT fell within Milan criteria came out in excellent prognosis: expected 4-year survival rate of 85% and an HCC recurrence–free survival rate of 92% after LT^3^,^12^. So, this study employed Milan criteria to grade our cases. We also utilized PSM to reduce selecting biases, particularly for those risk factors once proved to associate with poorer outcomes like AFP level, tumor mass and Milan criteria, etc. In the past researches, however, grading was not all based on Milan criteria. Moreover, since most preoperative scoring was only based on radiological images, it added to the potential bias of underestimation. It has been reported that underestimation of Milan criteria happened from time to time^13^,^14^. In this study, all those scorings were validated by measurement on dissected samples, which added accuracy to the grading.

Chronic HBV infection is the most common predisposed liver diseases in Chinese HCC patients, with 100-fold more likely to develop this malignancy^15^. The prestransplantation HBV-infectious rate is fairly high at our center at 92.89% entirely, respectively 89.29% in SLT population and 93.36% in PLT population, which was comparable with each other. We failed to find an association of either HBV infection history or positive tilter of virus DNA before transplantation with the increasing risk of tumor recurrence. This absence of correlation may either lie in lack of HBV negative patient group, or potent and regular anti-virus treatment after transplantation.

As for salvage transplantation itself, in the past 5 years, many articles, meta-analysis and reviews were published focusing on this topic. PLT was proved to be significantly associated with better OS or RFS only in 4 researches^8^,^16^,^17^,^18^. However, most studies showed a literally improved OS or RFS for PLT rather than SLT^6^,^7^,^9^,^19^,^20^,^21^,^22^,^23^. Dispute might lie in the heterogeneous nature in SLT definition or process. First, in some researches with optimistic results, later SLT was performed as a rescue of deteriorating liver function rather than recurrence^8^. A recent research has verified that de principe SLT has greater OS or RFS than LT performed following recurrence diagnosis^24^. We therefore narrow the recruiting standard of SLT in this study to HCC recurrence only. Second, the survival was either calculated from the point of LR or SLT^7^,^8^,^20^,^22^,^23^,^25^. Though, with consideration of all-dimensional analysis of patients’ prognosis, the intention-to-treat model was employed by some researches, a calculation based on individual survival can’t reflect the utilization efficacy of every donor. In order to better serve our research purpose, we chose to calculate OS or RFS from the final transplantation surgery.

It has been reported that the presence of microvascular invasion increased hazard ratios of recurrence in both resection and liver transplantation^12^,^26^. Due to the absence of more detailed pathological information, this factor was pitifully omitted from this research.

Finally, since this research adopted a retrospective per-protocol model, it inevitably had the risk of selection bias. A future prospective study targeting larger samples is expected to give more reliable conclusion.

## Conclusion

In conclusion, this study demonstrates that HCC recurrence and survival after SLT is significantly inferior to after PLT. These findings go against the common realized concept that SLT is as good as PLT. A wide feasibility of SLT should be given a second consideration.

## Additional Information

**How to cite this article**: Shan, Y. *et al*. Salvage Liver Transplantation Leads to Poorer Outcome in Hepatocellular Carcinoma Compared with Primary Liver Transplantaion. *Sci. Rep.* **7**, 44652; doi: 10.1038/srep44652 (2017).

**Publisher's note:** Springer Nature remains neutral with regard to jurisdictional claims in published maps and institutional affiliations.

## Figures and Tables

**Table 1:** Patient characteristics before and after propensity score matching (PSM).

Objects	Entire patients (n = 239)			PSM (23 pairs)		
	SLT	PLT	*p*	SLT	PLT	*p*
Age (y) Mean ± SD	47.79 ± 8.64	50.45 ± 9.24	0.149	47.70 ± 9.14	50.35 ± 7.61	0.572
Gender Male/female	24/4	179 /32	1	19/4	21/2	0.665
AFP (mg/mL) median (IQR)	166.2 (13.47~398.20)	107.7 (14.08~397.80)	0.714	170.0 (22.70~522.40)	166.2 (20.80~502.80)	0.982
CA19–9 (U/mL) median (IQR)	20.40 (8.50~37.15)	36.7 (18.60~56.90)	<0.001	20.4 (7.40~33.20)	36.70 (12.17~45.27)	0.141
Tumor status			0.548			0.238
Within Milan No. (%)	13 (46.43)	113 (53.55)		9 (39.13)	14 (60.87)	
Over Milan No. (%)	15 (53.57)	98 (46.45)		14 (60.87)	9 (39.13)	
HBsAg (+) No. (%)	25 (89.29)	199 (94.31)	0.395	21 (91.30)	21 (91.30)	1.000
HBV DNA (+) No. (%)	9 (32.14)	133 (63.03)	0.001	8 (39.13)	13 (43.48)	0.236
HCV (+)	1 (3.57)	4 (1.90)	0.467	1 (4.35)	1 (4.35)	1
Child-Pugh No. (%)			0.026			0.089
A	17 (60.71)	76 (36.02)		16 (69.57)	9 (39.13)	
B	6 (21.43)	97 (45.97)		5 (21.74)	12 (52.17)	
C	5 (17.86)	38 (18.01)		2 (8.70)	2 (8.70%)	
Rapamicin (+) No. (%)	14 (50%)	86 40.76%	0.416	14 (60.97)	12 (52.17)	0.767

**Table 2:** Multivariate analysis for recurrence-free survival (RFS) and overall survival (OS) rates before PSM.

Variables	RFS		OS	
	HR (95% CI)	*p*-value	HR (95% CI)	*p*-value
Age (y)	1.01 (0.97~1.05)	0.783	1.03 (0.99~1.07)	0.104
Gender (female)	0.35 (0.90~1.11)	0.750	0.63 (0.25~1.57)	0.299
Donor type split	5.48 (0.16~192.38)	0.349	5.89 (0.20~170.03)	0.301
AFP (ng/mL)	1.001 (1.000~1.001)	<0.001	1.001 (1.000~1.001)	0.006
CA19–9 (U/mL)	1.00 (0.997~1.004)	0.502	1.00 (1.00~1.00)	0.658
HBsAg (+)	2.03 (0.37~11.11)	0.414	0.72 (0.16~3.27)	0.671
HBV DNA (+)	1.16 (0.52~2.56)	0.712	0.79 (0.39~1.57)	0.497
HCV (+)	0.75 (0.06~10.01)	0.824	1.16 (0.14~10.0)	0.890
Tumor mass (cm)	1.12 (1.02~1.23)	0.023	1.11 (1.01~1.22)	0.040
Tumor number	0.96 (0.70~1.33)	0.843	1.20 (0.89~1.63)	0.239
Tumor status within Milan	0.21 (0.07~0.59)	0.003	0.29 (0.12~0.68)	0.004
Rapamicin (+)	0.85 (0.52~1.39)	0.517	1.30 (0.64~2.65)	0.469
Child-Pugh A	1.18 (0.22~6.50)	0.847	0.71 (0.17~3.00)	0.636
Child-Pugh B	2.06 (0.53~8.06)	0.300	1.49 (0.46~4.76)	0.506

**Table 3:** Multivariate analysis for recurrence-free survival (RFS) and overall survival (OS) rates After PSM.

Variables	RFS		OS	
	HR (95% CI)	*p*-value	HR (95% CI)	*p*-value
Age (y)	1.05 (0.96~1.15)	0.265	1.09 (0.99~1.17)	0.064
Gender (female)	0.10 (0.00~7.48)	0.174	0.73 (0.55~1.37)	0.968
Donor type split	8.33 (0.91~76.92)	0.061	10.08 (0.70~145.81)	0.090
AFP (ng/mL)	1.001 (1.000~1.001)	<0.001	1.011 (1.002~1.021)	0.017
CA19–9 (U/mL)	1.00 (0.99~1.02)	0.480	1.000 (0.999~1.001)	0.853
HBsAg (+)	0.09 (0.00~1.76)	0.110	0.32 (0.01~7.74)	0.483
HBV DNA (+)	5.13 (0.83~31.44)	0.077	1.92 (0.40~9.19)	0.415
HCV (+)	9.52 (2.02~13.33)	0.924	1.26 (0.16~10.07)	0.840
Tumor diameter (cm)	1.26 (1.05~1.51)	0.013	1.28 (1.07~1.53)	0.006
Tumor number	0.75 (0.39~1.47)	0.753	0.98 (0.50~1.89)	0.945
Tumor status within Milan	0.08 (0.01~0.53)	0.009	0.27 (0.14~0.50)	<0.001
Rapamicin (+)	0.88 (0.15~5.01)	0.874	0.96 (0.18~5.10)	0.961
Child-Pugh A	0.01 (0.00~15.16)	0.203	0.77 (0.40~1.49)	0.443
Child-Pugh B	0.02 (0.00~12.12)	0.219	1.06 (0.57~1.98)	0.855

**Table 4:** Hazard ratio (HR) for clinical outcomes in the SLT group compared with the PLT group in propensity matched cohort.

Outcomes	Unadjusted		Multivariable adjusted		Adjusted by PSM	
	HR (95% CI)	*p*-value	HR (95% CI)	*p*-value	HR (95% CI)	*p*-value
OS	1.59 (0.90~2.82)	0.109	2.17 (1.18~4.01)	0.013	2.84 (1.06~7.67)	0.038
RFS	1.90 (1.15~3.16)	0.013	1.98 (1.05~3.72)	0.035	3.53 (1.02~12.20)	0.037
